# Nucleated red blood cells are a predictor of mortality in patients under extracorporeal membrane oxygenation

**DOI:** 10.1186/s40001-023-01243-y

**Published:** 2023-08-07

**Authors:** Sebastian Loesaus, Peter Konrad Zahn, Matthias Bechtel, Justus Thomas Strauch, Dirk Buchwald, Andreas Baumann, Dinah Maria Berres

**Affiliations:** 1 Department of Anaesthesiology, Intensive Care and Pain Medicine, Ruhr- University Hospital Bergmannsheil, Bürkle-de-la-Camp- Platz 1, 44789 Bochum, Germany; 2 Department of Cardiothoracic Surgery, Ruhr-University Hospital Bergmannsheil, Bürkle-de-la-Camp-Platz-1, 44789 Bochum, Germany

**Keywords:** Nucleated red blood cells, ECMO, Mortality, Intensive care unit

## Abstract

**Background:**

The presence of Nucleated Red Blood Cells (NRBCs) in critically ill patients is associated with higher mortality and poor prognosis. Although patients on extracorporeal support such as veno-venous or veno-arterial extracorporeal membrane oxygenation (VV/VA-ECMO) are severely ill, NRBCs have rarely been investigated regarding their predictive value so far.

**Methods:**

As part of a retrospective study, we examined all cardiothoracic surgery patients from July 2019 to September 2020 who received ECMO treatment during their inpatient stay. The aim of this study was to investigate the occurrence of NRBCs during ECMO support in terms of their predictive value for mortality.

**Results:**

In total 30 patients (age at admission: 62.7 ± 14.3 year; 26 male; ECMO duration: 8.5 ± 5.1 days; ICU duration: 18.0 ± 14.5 days) were included. 16 patients (53.3%) died during their inpatient stay. There were no significant differences in demographic characteristics between VA- or VV- ECMO patients. NRBCs occurred in all patients while under ECMO support. NRBC value was significant higher in those who died (2299.6 ± 4356.6 µl) compared to the surviving patients (133.6 ± 218.8 µl, *p* < 0.001). Univariate analysis found that patients with a cutoff value of ≥ 270 NRBCs/µl during ECMO support were 39 times more likely to die (OR 39.0, 95% CI 1.5–997.5, *p* < 0.001). 12 out of 13 patients (92.3%) with ≥ 270 NRBCs/µl died. The area under the curve (AUC) of the receiver operating characteristic curve was 0.85 (95% CI 0.69–0.96) with a sensitivity of 75.0% and a specificity of 92.9%.

**Conclusion:**

NRBCs appear to be an accurate biomarker for mortality in patients with ECMO support. They may be helpful in deciding if therapy becomes futile.

*Trial registration* DRKS00023626 (December 20th 2020).

**Supplementary Information:**

The online version contains supplementary material available at 10.1186/s40001-023-01243-y.

## Background

Patients on ECMO are severely ill patients. In literature, mortality data differ depending on ECMO mode and reason for therapy. According to the Extracorporeal Life Support Organization (ELSO) registry, the survival rate to discharge in adults is 49% (pulmonary 58%, cardiac 45%, ECPR 30%) [1]. With the beginning of an extracorporeal membrane oxygenation there are multiple pre-tests to evaluate risk factors and estimate the probability of death like SAVE-, RESP-, REMEMBER- and ENCOURAGE Score [2–5]. The risk of mortality for patients undergoing cardiothoracic surgery is estimated with EUROScore II [6]. Unfortunately scores for controlling any kind of ongoing process are still missing. With measuring values such as lactate and bilirubin it is possible to catch hints of organ failure [7, 8]. NRBCs are not found in the peripheral blood of healthy adults. In healthy newborns, NRBCs can be found in the peripheral blood up to five days after birth [9]; in case of premature birth or fetal hypoxia NRBCs can still be found after that period [10]. They are early erythrocyte precursor cells and their presence in peripheral blood is an indicator for either increased erythropoiesis or a defect bone marrow barrier. Regardless of the mechanism, the cause is always stress like hypoxemia, sepsis, or malignancy [11]. The release and secretion of NRBCs is stimulated by Interleukin 3, Interleukin 6 and Erythropoietin [12]. In addition, these cytokines are also released as part of an inflammatory response on ECMO therapy [13].

The presence of NRBCs in the peripheral blood has been described as being associated with mortality in several different populations of patients. Schwartz et al. already described significant correlation between occurrence of NRBCs in peripheral blood and increased mortality back in the 1950’s [14]. Stachon et al. previously depicted the occurrence of NRBCs and their predictive power in cardiothoracic surgery patients [15]. Lehnhardt et al. described the same in burn patients [16]. In addition, the presence of NRBCs was shown to increase mortality even after discharge. It also had a negative predictive effect on hospital readmissions [17].

In 2021, Piggot et al. first demonstrated that NRBCs in neonates and children < 1 year of age treated with VA—ECMO are a useful marker for mortality [18]. To the best of our knowledge there are currently no studies available that have investigated the occurrence and prognostic significance of NRBCs in adult patients while on ECMO except for a subgroup analysis in patients with ARDS [19]. Not to be neglected, however, is the question if NRBCs can still be seen as a mortality predictor in patients with known hematological disorder [20].

The aim of our study was to investigate the occurrence and levels of NRBCs in cardiothoracic patients on ECMO in terms of their prognostic significance regarding patient mortality.

## Methods

### Ethical aspects, study population

The present study was performed at the University Hospital Bergmannsheil Bochum (which is a registered ECMO center). Retrospectively, the data of 30 patients of our intensive care unit were examined regarding NRBC levels. Blood samples were taken daily at the same point of time in patients with extracorporeal support procedures. 30-day-mortality after decannulation was investigated. Most patients had undergone cardiac surgery (Coronary artery bypass grafting, Valve or Aortic surgery) except for two patients who were treated conservatively for pulmonary embolism. Exclusion criteria were age under 18 years, ECMO duration less than 24 h and patients with history of hematologic disease.

After obtaining permission from the local ethics committee (No.: 20–706430), the study was registered in the German clinical Trial register (German Clinical Trials Register-No: DRKS00023626, retrospectively registered, December 20th 2020, https://www.drks.de/drks_web/navigate.do?navigationId=trial.HTML&TRIAL_ID=DRKS00023626). No informed consent for retrospective, anonymous data are required and informed consent was waived by the ethics committee.

Data were collected in the period from July 2019 to September 2020 from the in-house patient data management system (Medico, Cerner, Germany).

All patients were treated with the following ECMO systems: Cardiohelp, Rotaflow (Getinge Group) and Novalung (Fresenius Medical Care). Cannulation strategies were chosen regarding the physicians’ preference and the clinical setting such as femoral or axillary arterial and femoral or internal jugular venous cannulation.

### Laboratory tests

The blood samples were taken daily with a commercially available EDTA tube. The determination of NRBCs was performed with the UniCel DxH 600—Coulter Cellular Analysis System (Sysmex, Kobe, Japan). Counts are reported in NRBC cells (n)/µl.

### Statistical analysis

Categorical characteristics are shown as total and percentage and compared using Exact Fisher’s test. Metric characteristics are described based on mean, median, standard deviation, interquartile range and minimum and maximum compared using the Mann–Whitney *U* test. Level of significance was assumed *p* < 0.05. To determine the cutoff, we used the Youden-Index as well as Liu’s method and Nearest-method. Odd`s ratio as probability of mortality was calculated. NRBCs discriminatory power was assessed by calculating the area under the receiver operating characteristic (ROC) curve. To visualize the survival, we used the Kaplan—Meier curve. Stata/IC 16.1 software (StataCorp 4905 Lakeway Drive College Station, TX 77845 USA) was used for analyses.

## Results

A total of 30 patients were included in the analysis. Mean age was 62.7 years (± 14.3). The study group consisted of 26 men and 4 women.

All patients were in a life-threatening condition and needed extracorporeal life support either as veno-venous- (VV) or veno-arterial- (VA) device for more than 24 h. A number of 12 patients was treated with VV-ECMO, 18 patients needed a VA-ECMO. Additionally, 3 out of 30 were supported with an intra-aortal balloon pump (IABP), 4 others had a mechanical ventricular support (MVS). In our institution, we use the IMPELLA-family (Abiomed, Danvers, MA). There were no statistic significant differences regarding mortality when IABP (*p* = 1.000) or MVS (*p* = 0.336) was used.

All patients underwent cardiac surgery except for two patients suffering from pulmonary embolism who were treated conservatively. The cause for device implantation was multifactorial in a lot of patients so that multiple mentions are possible. Most patients were in an acute life-threatening condition: under CPR (10), post—cardiotomy (10), unbalanced gas exchange (10), right heart failure (5) and pulmonary embolism (2). Reasons for ECMO therapy and mode of operation as well as baseline characteristics, laboratory values and demographics are shown in Table 1. Of these 30 patients 14 survived a 30-day-follow-up after decannulation. While still on ECMO 13 out of 16 patients died. 3 patients died shortly after decannulation. Metric characteristics showed no significant differences between survivors and non survivors.

**Table 1:** Demographics and clinical data

Variable	*n*	Survivors	Deceased	*p*-value
Patients	30	14 (46.7%)	16 (53.3%)	–
Gender				
Male	26	12 (46.2%)	14 (53.8%)	
Female	4	2 (50.0%)	2 (50.0%)	1.000
Age	30	59.6 (± 16.9)	65.4 (± 11.4)	0.479
BMI kg/m^2^	30	30 (± 5.5)	29.2 (± 5.3)	0.662
Causes requiring ECMO^b^				
RV failure	5	3 (60.0%)	2 (40.0%)	0.642
Cardiac arrest	12	5 (41.7%)	7 (58.3%)	0.722
PCS	10	3 (30.0%)	7 (70.0%)	0.260
PAE	2	2 (100%)	0 (0%)	0.209
Respiratory failure	10	6 (60.0%)	4 (40.0%)	0.442
Device characteristics				
VA-ECMO	18	7 (38.9%)	11 (61.1%)	
VV-ECMO	12	7 (58.3%)	5 (41.7%)	0.457
ECMO Days	30	7.4(± 4.1)	9.4(± 5.7)	0.287
Blood flow/day 1	30	3.5(± 0.7)	4.4 (± 0.4)	0.001^a^
Sweep flow/day 1	30	2.1(± 0.8)	2.8 (± 0.9)	0.021^a^
Comorbidities				
Hypertension	23	9 (39.1%)	14 (60.9%)	0.204
Smoking	12	5 (41.7%)	7 (58.3%)	0.722
Diabetes	6	4 (66.7%)	2 (33.3%)	0.378
COPD	3	0 (0%)	3 (100%)	0.228
Chronic renal failure	7	2 (28.6%)	5 (71.4%)	0.399
Hyperlipidemia	13	4 (30.8%)	9 (69.2%)	0.159
Type of surgery				
Isolated CABG	11	4 (36.4%)	7 (63.6%)	0.466
Isolated valve	7	3 (42.9%)	4 (57.1%)	1.000
CABG & valve	7	5 (71.4%)	2 (28.6%)	0.204
Aortic repair	1	0 (0%)	1 (100%)	1.000
Embolectomy	1	1 (100%)	0 (0%)	0.467
Pericardectomy	1	0 (0%)	1 (100%)	1.000
Euroscore II	30	20.2 (± 19.0)	27.3 (± 22.3)	0.339
RESP score	12	2.9 (± 1.3)	0.2 (± 1.6)	0.017^a^
SAVE score	18	− 6.9 (± 5.0)	− 9.7 (± 3.4)	0.456
SAPS II	30	43.6 (± 11.3)	54.2 (± 11.0)	0.010^a^
Laboratory values at ECMO initiation				
CRP (mg/dl)	30	6.9 (± 7.8)	8.7 (± 10.1)	0.675
Platelets (/nl)	30	196 (± 147.4)	166.8 (± 111.8)	0.637
WBC (/nl)	30	19.8 (± 6.9)	15.9 (± 6.2)	0.103
Bilirubin (mg/dl)	30	2.0 (± 0.9)	1.9 (± 2.7)	0.057
GFR (mg/dl)	30	54.3 (± 26.2)	52.4 (± 27.6)	0.884
Hemoglobin (mg/dl)	30	10.6 (± 2.3)	10.0 (± 0.7)	0.910
Lactate (mmol/l)	30	5.5 (± 2.8)	7.2 (± 3.9)	0.308
NRBCs (/µl)	30	24.6 (± 39.8)	108.2 (± 205.4)	0.170
Lactate (mmol/l)24 h after ECMO initiation	30	1.9 (± 1.1)	4.4 (± 3.4)	< 0.001^a^
pRBCs (n)	30	23.1 (± 20.9)	22.9 (± 15.8)	0.803

Data presented as n (%) or mean ($\pm$ SD), categorial variables were analyzed with Fisher’s exact test. Metric variables were analyzed with the Mann–Whitney U test

*BMI* Body mass index, ECMO extracorporeal membrane oxygenation, *PCS* post cardiotomy Syndrom, *PAE* pulmonary arterial embolism, *V-A* veno – arterial, *V-V* veno – venous, *COPD* chronic obstructive pulmonary disease, *CABG* coronary artery bypass grafting, *RESP Score* Respiratory Extracorporeal Membrane Oxygenation, Survival Prediction Score, *SAVE Score* Survival After Veno-arterial Extracorporeal membrane oxygenation Score, *SAPS II* simplified acute physiology score, *CRP* c-reactive protein, *WBC* white blood cell, *GFR* glomerula filtration rate, *NRBCs* nucleated red blood cells, *pRBCs* Packed red blood cells

^a^*p* < 0.05

^b^multiple mentions possible

### NRBCs as mortality predictor

**Fig. 1:**
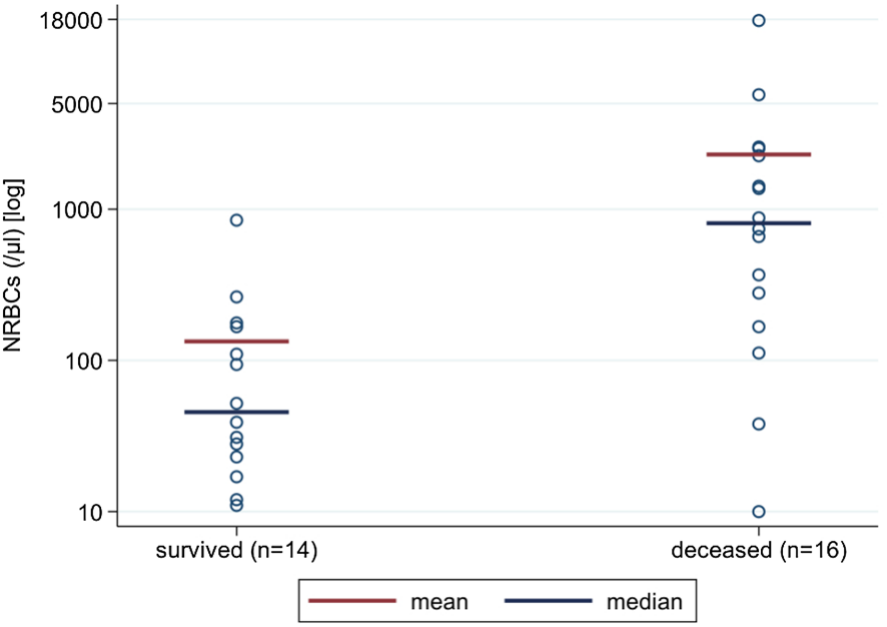
NRBCs concentrations found in peripheral blood of patients who either died or survived ECMO procedures

**Fig. 2:**
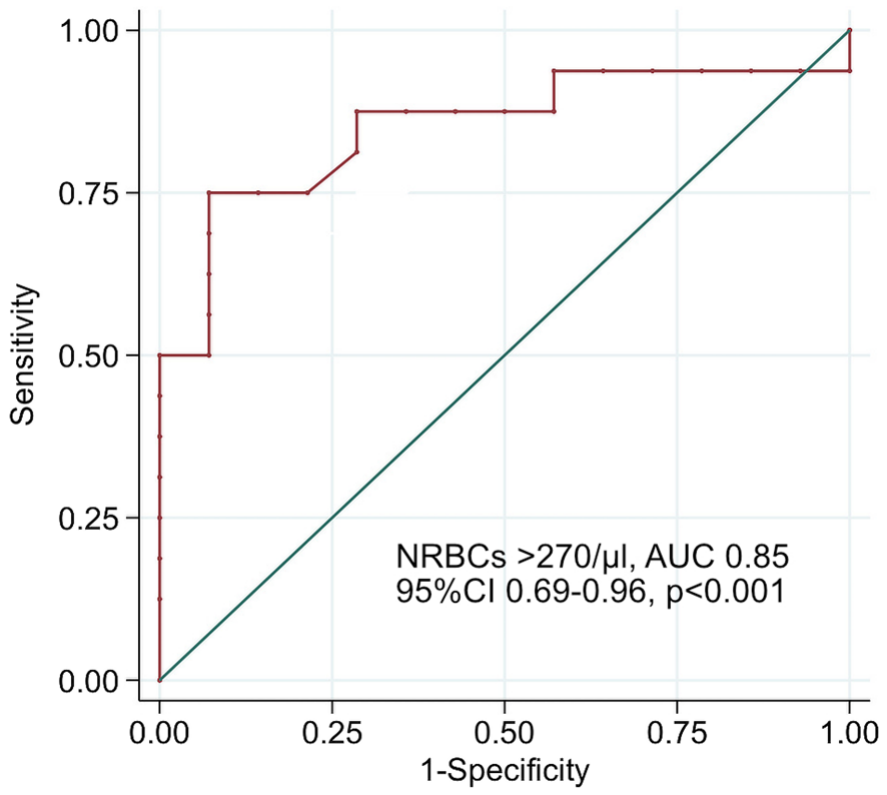
Area under the receiver operating characteristic curve (AUROC) for NRBC count ≥ 270 µl as predictor of mortality while on ECMO

**Fig. 3:**
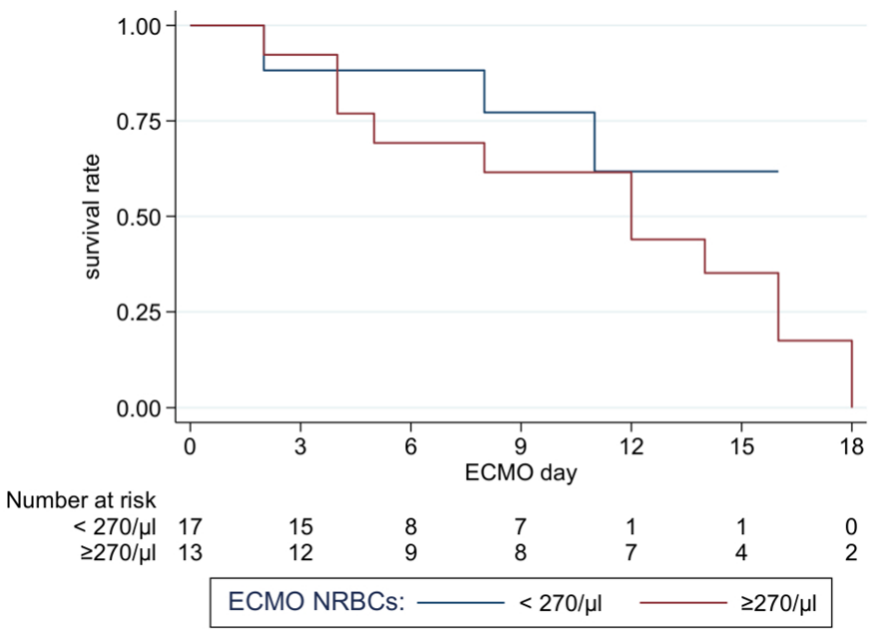
Kaplan—Meier survival curve of ECMO patients according to NRBCs count

NRBC values were higher in those who died (2299.6 ± 4356.6 µl) compared to those who survived (133.6 ± 218.8, *p* < 0.001) (Table 2, Fig. 1). We used the Youden-Index as well as Liu’s method and “Nearest method” to define the best cutoff for mortality prediction. All three statistical procedures defined NRBC count with more than 270 µl on at least one day on ECMO as significant. 13 patients reached or exceeded this cutoff, 12 (92.3%) of these 13 patients died. In contrast, patients below this cutoff showed a significant lower mortality [4 (23.5%) of 17, *p* < 0.001]. Patients with a cutoff value of ≥ 270 NRBCs/µl during ECMO support were 39 times more likely to die (OR 39, 95% CI 1.52–997.54, *p* < 0.001) (Table 3). ROC curve evaluating NRBCs as a predictor of mortality, showed an AUC of 0.85 (95% CI 0.69–0.96, *p* < 0.001), with a sensitivity of 75% and specificity of 92.9%, and positive and negative predictive values of 92.3% and 76.5%, respectively (Fig. 2). Kaplan–Meier curve shows the overall survival (Fig. 3). Additional File 1 Figure S1 shows the distribution of NRBC/µl over time.

**Table 2:** NRBCs count(/µl) during ECMO

	*n*	Mean	SD	Median	IQR	Min–Max	*p*-value^a^
Survivors	14	133.6	218.8	45.5	23–167.0	11–846.0	
Deceased	16	2299.6	4356.6	808.0	223–2389.0	10–17682.0	< 0.001^a^

^a^Mann-Whitney-U-test

**Table 3:** Mortality odds ratio (95%CI)

NRBCs count(/µl)	*n*	Survivors	Deceased	*p*-value^a^ OR [95%-CI]
< 270	17	13 (76.5%)	4 (23.5%)	< 0.001^a^
≥ 270	13	1 (7.7%)	12 (92.3%)	39.00 (1.52–997.54)

^a^Exact Fisher test

## Discussion

ECMO treatments are predominantly high-risk, high-cost, and often ethically debatable [21–23] therapeutic options. During installation as well as during therapy, several complications may occur such as hemorrhage, limb ischemia, neurological damage, liver, or kidney failure [24–26]. At the same time, these procedures provide physicians with a tool to support patients with either respiratory or circulatory failure.

The occurrence of NRBCs in the peripheral blood is pathophysiological due to a higher erythropoietic pressure and the failure of the spleen to clear these precursor cells. Since circumstances such as systemic inflammatory reactions, hypoxia, massive hemorrhage, and cardiac failure increase erythropoietic pressure, the appearance of NRBCs indicates a disturbance of homeostasis [17]. Although the exact mechanism is not clear yet, the appearance of these progenitor cells can thus be taken as an indicator of ongoing organ failure. It is therefore not surprising that, in contrast to other studies, we were able to detect NRBCs in the peripheral blood of all our patients, which reflects the severity of our patients' diseases. However, despite the fact, that no causality can be inferred from a retrospective observational study, the relationship between the occurrence of NRBCs and the higher probability of death is plausible. Thus, our results support the context of other previous studies, which have shown that NRBCs can serve as prognostic markers.

Stachon et al. pointed out the prognostic significance in patients who underwent cardiothoracic surgery [15]. Lehnhardt et al. showed the same in burn patients. In their study, the sole presence of NRBCs—irrespective of the number of cells—was a predictor of mortality with an Odd’s ratio of 8.3 (CI 95% 4.5–15.3) [16]. Even at the time of admission in the emergency department higher blood levels of NRBCs seem to be associated with higher mortality [27]. Furthermore, for post discharge outcome in critically illness survivors NRBC counts seem to have a prognostic value. Odds’ ratio for 90-day-mortality post-discharge in patients with more than 200 µl NRBCs was 3.72 (95% CI 2.16–6.39) [17].

In our study, patients in the acute phase of illness with cardiocirculatory and/ or respiratory failure requiring ongoing extracorporeal support were studied. Piggott et al. demonstrated an NRBCs’ increase greater than 50% after ECMO decannulation in children was associated with higher inpatient mortality (OR 17.1, 95% CI 3.1–95.1, *p* = 0.001) [16]. Therefore, our results fit closely with those of the limited number of previous studies.

When evaluating the cutoff for NRBCs we saw a 39 times greater risk for death when NRBCs reached or passed 270 µl. Furthermore, the ROC curve of NRBCs under ECMO therapy reached an AUC of 0.85. In addition, significantly higher levels of nucleated red blood cells were detected in the blood of the deceased patients. In summary, all this indicates NRBCs as a very strong predictor for mortality in critically ill patients independent of the ECMO mode. Besides, it is an advantage that NRBCs can be routinely determined every day. It is easily available and low of cost.

As a part of future investigations, we would like to incorporate NRBC count, respectively, a defined cutoff in a clinical mortality score. There are some existing mortality scores like EuroSCORE II for cardiothoracic patients [6], RESP Score for patients undergoing VV-ECMO [3] or SAVE Score for those with VA-ECMO [2], just to name a few of them. Some of them are used to estimate whether a device should be implemented or not, most scores are ascertained for one time before implementation based on pre ECMO values and, therefore, not flexible [28]. None of these ECMO scores, nor any of the intensive care scores such as Apache II or SAPS II, despite their good prognostic strength, include the incidence and level of NRBCs [29]. In the past there have been a few attempts to integrate NRBCs e.g., in APACHE II with reliable results [30, 31]. Stachon et al. found for their modified APACHE II score an area under curve about 0.91 compared to 0.87 and 0.72 for the APACHE II and NRBCs alone [30]. To validate such a modified risk score further prospective studies are needed.

Even though we only included a small number of cases in our study, there is a very strong trend that this parameter is suitable to measure mortality in the context of ECMO therapy.

With a routine daily review of NRBCs implemented into a scoring system, perhaps a flexible tool could be created to evaluate the patients’ process and give physicians a chance to take a closer look at the patient before further deterioration occurs. While further large prospective studies have yet to demonstrate whether NRBCs can serve as a marker to detect mortality independent of ECMO mode, based on our results, we can say that while NRBCs are not new in business, they appear to be old but gold.

## Conclusion

In our study group, we were able to show that the level of nucleated red blood cells is an excellent marker for predicting mortality on ECMO. Of course, diagnosis and treatment decisions in the context of ECMO procedures should never be based on one laboratory value, but decision making in the context of a scoring system in the ethical dilemma "is the patient still alive or am I just keeping him alive" should be the goal for future studies. In terms of its prognostic significance, such a score should include the level and progression of NRBCs.

## Limitations

There are several limitations of our study. Due to the retrospective design of our study and the small number of patients further studies are necessary. Prospective studies might also be able to reveal the mechanism(s) behind the observed association. Also, we investigated 26 male and only 4 female patients. We assume that the prognostic value for both genders is the same.

### Supplementary Information

**Additional file 1. Figure S1 **Distribution of NRBCs/μl over time

## Data Availability

The datasets used and/or analyzed during the current study are available from the corresponding author on reasonable request.

## Abbreviations

BMI: Body Mass Index
CABG: Coronary artery bypass grafting
CI: Confidence interval
COPD: Chronic obstructive pulmonary disease
CPR: Cardiopulmonary resuscitation
CRP: c-reactive protein
ECMO: Extracorporeal membrane oxygenation
eCPR: extracorporale cardiopulmonary reanimation
ELSO: Extracorporeal life support organization
GFR: Glomerula filtration rate
ICU: Intensive care unit
IQR: Interquartile range
M: Mean
NRBCs: Nucleated red blood cells
OR: Odds ratio
PCS: Post cardiotomy syndrome
PAE: Pulmonary arterial embolism
pRBCs: Packed red blood cells
RESP Score: Respiratory extracorporeal membrane oxygenation survival prediction score
SAPS II: Simplified acute physiology score
SD: Standard deviation
SAVE Score: Survival after veno-arterial extracorporeal membrane oxygenation score
V-A: Veno - arterial
V-V: Veno - venous
WBC: White blood cell count
